# Chemistry, Occurrence, Properties, Applications, and Encapsulation of Carotenoids—A Review

**DOI:** 10.3390/plants12020313

**Published:** 2023-01-09

**Authors:** Marco Antonio González-Peña, Ana Eugenia Ortega-Regules, Cecilia Anaya de Parrodi, José Daniel Lozada-Ramírez

**Affiliations:** 1 Departmennt of Chemical, Food and Environmental Engineerig, Universidad de las Américas Puebla, Cholula, Puebla 72810, Mexico; 2 Department of Health Sciences, Universidad de las Américas Puebla, Cholula, Puebla 72810, Mexico; 3 Department of Chemical and Biological Sciences, Universidad de las Américas Puebla, Cholula, Puebla 72810, Mexico

**Keywords:** carotenoids, antioxidants, vitamin A, bioavailability, stability, carotenoid protection

## Abstract

Carotenoids are natural lipophilic pigments and antioxidants that are present in many fruits and vegetables. The consumption of carotenoids is correlated with positive health effects and a decreased risk of several chronic diseases. Provitamin A carotenoids (β-carotene, α-carotene, γ-carotene, and β-cryptoxanthin) are essential for the development and maintenance of sight. β-carotene, α-carotene, zeaxanthin, β-cryptoxanthin, lutein, and lycopene have high antioxidant activity and promote free radical scavenging, which helps protect against chronic diseases. However, carotenoids are chemically unstable and prone to oxidation in the presence of light, heat, oxygen, acids, and metal ions. The use of carotenoids in the food industry is limited due to their poor solubility in water, bioavailability and quick release. Encapsulation techniques, such as microencapsulation, nanoencapsulation and supercritical encapsulation, are used to overcome these problems. The objective of this paper is to describe the characteristics and potential health benefits of carotenoids and advances in encapsulation techniques for protecting and enhancing their solubility or bioavailability.

## 1. Introduction

Carotenoids are a group of pigments found in fruits, flowers and vegetables, such as tomato, carrot, pineapple, papaya, marigold flower, sunflower, annatto, saffron, and green leaves. They are responsible for yellow, orange, and red colors in plants, and are used commercially as natural colorants and ingredients in nutritional supplements [1].

In recent years, studies of plant pigments have increased in importance, given their provitamin A activity, and they have been classified as natural antioxidants and bioactive compounds. Studies have shown evidence of their role in the prevention of chronic degenerative diseases, cardiovascular diseases, cancer, macular degeneration and cataract formation [2]. Carotenoids are involved in immune system modulation and cell communication, embryonic development, hematopoiesis and apoptosis, and possess antioxidant, anti-inflammatory, anti-angiogenic and antiproliferative properties [3,4].

However, the use of carotenoids in the food industry is restricted due to their poor water solubility, low bioavailability, chemical instability and high sensitivity to oxidation in multiple environmental conditions, such as heat, light, oxygen, acids and metal ions [5]. To overcome this inconvenience, encapsulation techniques have been used to improve the stability, solubility and bioavailability of carotenoids. Encapsulation has been used in the food industry for more than 60 years to cover food ingredients, enzymes, cells or other functional compounds in small capsules, to protect them from environmental conditions, increase their shelf life, or to mask component attributes such as undesirable flavors [6].

Therefore, the objective of this paper is to provide an overview of the nature and characteristics of carotenoids, their use in food and medicine as antioxidants and health promoters, and encapsulation techniques employed to protect them from degradation and to improve their bioavailability and solubility.

## 2. Chemistry of Carotenoids

Carotenoids are a group of pigments, mostly of plant origin, responsible for the yellow, orange and red colors in fruits and vegetables. All have antioxidant activity, and some are precursors of vitamin A. Moreover, carotenoids have a role in intercellular communication, immune system activation and disease prevention [3,7], and hence they promote human health.

Carotenoids comprise eight repetitive units of isoprene, with cyclic or linear structures at both ends of the carbon chains, resulting in multiple cis and trans isomers, with the latter being more abundant in nature [8,9]. Carotenoids are classified into carotenes and xanthophylls. Carotenes, such as α-carotene, β-carotene, γ-carotene, and lycopene [8], are highly soluble in organic solvents and insoluble in polar solvents. In contrast, xanthophylls are soluble in polar solvents (e.g., alcohols) and organic solvents (e.g., ether and hexane). They are oxygenated derivatives of carotenes, forming alcohols, aldehydes, ketones, and acids. Examples of xanthophylls are fucoxanthin, lutein and violaxanthin [8,9]. Figure S1 shows the chemical structures of several carotenes and xanthophylls.

Carotenoids are stored in plant tissues (plastids), such as chromoplasts (colored plastids), amyloplasts (starch storage plastids) and elaioplasts (lipid storage plastids). In fruits, flowers and roots, carotenoids are located in the chromoplast, whereas in grains and oilseeds they are located in amyloplasts and elaioplasts, respectively [10]. In contrast, xanthophylls are freely found in green plant tissues, whereas in fruits and flowers they are found as esters of fatty acids [11].

**Figure 1:**
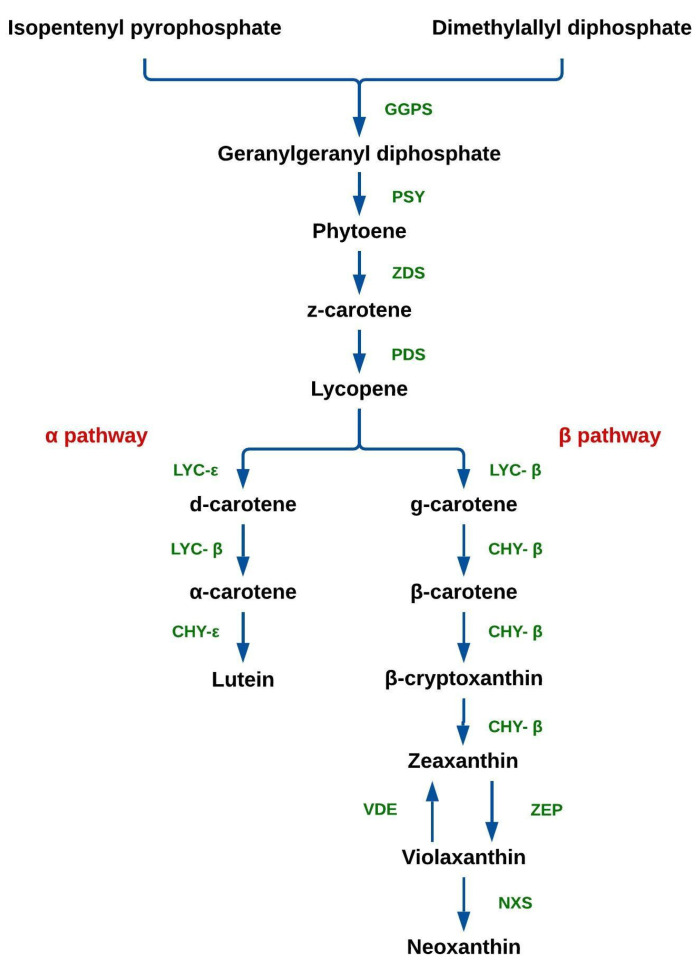
Carotenoid biosynthesis pathway in plants. Geranyl-geranyl pyrophosphate synthase (GGPS), phytoene synthase (PSY), ζ-carotene desaturase (ZDS), phytoene desaturase (PDS), lycopene ε-cyclase (LYC-ε), lycopene β-cyclase (LYC-β), α-carotene ring-ε hydroxylase (CHY-ε), β-carotene hydroxylase (CHY-β), zeaxanthin epoxidase (ZEP), violaxanthin de-epoxidase (VDE), and neoxanthin synthase (NXS).

The biosynthesis of carotenoids (Figure 1) takes place in the chloroplasts. Two molecules of geranylgeranyl diphosphate (GGPP) are produced from isopentenyl pyrophosphate (IPP) and dimethylallyl diphosphate (DMAPP), catalyzed by geranylgeranyl pyrophosphate synthase (GGPS). After this, two molecules of GGPP are condensed into phytoene by phytoene synthase (PSY). Subsequently, phytoene is desaturated into lycopene by ζ-carotene desaturase (ZDS) and phytoene desaturase (PDS). Lycopene is cyclized into α-carotene (α pathway) and β-carotene (β pathway), in reactions catalyzed by lycopene-ε (LYC-ε) and β-cyclase (LYC-β), respectively. Xanthophylls are synthesized from carotenes; lutein is formed by the action α-carotene ring-ε hydroxylase (CHY-ε) via the α pathway; β-carotene is converted into β-cryptoxanthin via the β pathway, in a reaction catalyzed by β-carotene hydroxylase (CHY-β), which also catalyzes its conversion into zeaxanthin. In turn, zeaxanthin can be converted into violaxanthin by zeaxanthin epoxidase (ZEP); conversely, violaxanthin can be converted into zeaxanthin by violaxanthin de-epoxidase (VDE). Finally, violaxanthin is converted into neoxanthin by neoxanthin synthase (NXS) [5,10,12].

## 3. Natural Occurrence of Carotenoids

More than 700 carotenoids have been identified, yet only 40 are included in the human diet, with β-carotene, α-carotene, lycopene, β-cryptoxanthin, lutein, and zeaxanthin the most common [13]. Carotenoid intake comes primarily from foods of plant origin (fruits and vegetables). α- and β-carotene are usually found in carrot, orange, pumpkin, pepper, sweet potato, mango, and vegetable leaves, varying in color from yellow, orange, and red. Lycopene imparts a bright red color to food, and is the main carotenoid in tomatoes, although it is also present in watermelon, guava, and papaya. β-cryptoxanthin is found in citrus fruit, melon, potato, guava and apple, giving them yellow and orange colors. The isomers lutein and zeaxanthin are found together naturally in yellow corn and marigold flower, although they are also found in broccoli, pumpkin, pepper, vegetable leaves, seeds and legumes. The xanthophylls violaxanthin (yellow), capsanthin, and capsorubin (orange to red) are frequently found in paprika and pepper. Neoxanthin, characterized by its yellow color, is a natural component of vegetable leaves. Bixin is the main component of annatto and is responsible for its characteristic red-brown color. Crocin is responsible for the yellow coloration of saffron [3,8,9,10].

Some carotenoids are found only in algae and seafood. Astaxanthin is responsible for the pink-red color of shrimp, salmon, and flamingo feathers, as it is naturally found in krill and microalgae haematococcus pluvialis, which are consumed by small crustaceans (shrimp and crawfish), fish (salmon), and birds (flamingo) [14,15], in that order. Fucoxanthin has a characteristic brown color and is only found in microalgae and the chloroplasts of brown algae [16].

The most abundant carotenoids in the Western diet include β-carotene and α-carotene from carrot, pumpkin, apricot, pepper, mango, coriander and spinach [3,13,17,18]; lutein from broccoli, pumpkin, spinach, corn, mango and papaya [3,13]; and lycopene from tomato, guava, papaya and watermelon [3,13,17,18,19,20].

Britton and Khachik (2009) proposed a ranking of fruits and vegetables based on their carotenoid levels, grouping the foods into the following categories: low level (0–1 µg/g), moderate level (1–5 µg/g), high level (5–20 µg/g), and very high level (>20 µg/g). Table S1 lists the carotenoid levels of some common fruits and vegetables consumed in America.

Carotenoid composition and content in food are affected by many factors, such as those inherent to the plant (variety, genotype and ripening stage), external to the plant (harvest season, growth conditions, post-harvest treatment, handling, storage conditions, plant diseases, and climatic conditions) [3,13].

## 4. Bioavailability of Carotenoids

Carotenoid bioavailability is defined as the fraction of carotenoid released from food that is absorbed in the intestine and made available for physiological processes or storage [13].

The nature of the food matrix containing carotenoids strongly affects their bioavailability. Due to their hydrophobic nature and location in plant tissues (plastids), carotenoid bioavailability in raw fruits and vegetables is limited. Therefore, carotenoids must be released from the cellular matrix and incorporated into a lipid fraction (micelles) during digestion to be absorbed [2].

Carotenoids are released from food mechanically by chewing, and chemically by the action of digestive enzymes (amylases, lipases, pepsin) and hydrochloric acid in the stomach [21]. These processes contribute to particle size reduction, thus increasing contact area for pancreatic lipases, bile salts, and enzymes, such as pancreatic amylases, nucleosidases, trypsinogen, chymotrypsinogen, carboxypeptidase, elastases, phospholipases, and carboxyl lipase ester, and the release of carotenoids and their incorporation into micelles [3,22]. Bile salts act as emulsifiers assisting with micelles formation and stabilization, whereas lipases break down lipids into fatty acids and monoglycerides, encouraging emulsification [22]. Micelles are absorbed into the enterocytes through passive diffusion or by binding to receptor proteins in the apical membrane for easy diffusion [2].

After absorption, carotenoids are enclosed in chylomicrons and transported to the bloodstream through the lymphatic system [7,17]. Once they have entered the bloodstream, the carotenoids are transported to the liver, where they are either stored or metabolized into vitamin A (only provitamin A carotenoids) by β-carotene binding oxygenases (BCO1 and BCO2). The rest are released back into the circulation and integrated into very low density (VLDL), low density (LDL), and high-density (HDL) lipoproteins to be distributed to other tissues, such as adipose tissue (vitamin A storage), skin and subcutaneous tissues (carotenes and xanthophylls reserve), macula lutea in the retina (lutein, zeaxanthin, and mesozeaxanthin), pancreas and vascular endothelium [22].

Carotenoid bioavailability is influenced by dietary factors, such as content and nature of carotenoids, fat content in the diet, and the interaction between carotenoids and other food components; and physiological factors, such as the rate of carotenoid absorption, nutritional state, genetic factors, and metabolism [2,17]. For instance, dietary fiber, especially soluble fiber, has been found to limit carotenoid availability as it affects the viscosity of the gastrointestinal content, size of lipid droplets, availability of bile salts, and enzymatic lipolysis of triglycerides [21]. Furthermore, Gul et al. [17] and Saini et al. [3] reported that the bioavailability of β-carotene in plants is low because carotenoids are bound to protein complexes, fibers, and cell walls, rendering them resistant to digestion and degradation, thus limiting their release. In contrast, several researchers have demonstrated the effect of minerals on the bioavailability of carotenoids. Borel et al. [23] found that the bioavailability of lycopene found in tomato paste is limited when ingested with 500 mg of calcium, although the mechanism is not completely understood, and micelle formation may be the limiting factor. Corte-Real et al. [24] found that the bioavailability of spinach carotenoids is not affected by 500 mg and 1000 mg of calcium.

Adding fat to the food improves the bioavailability of carotenoids as lipids favor micelle formation through the release of bile salts [2]. In this sense, Marriage et al. [25] showed that plasma concentrations of lycopene and zeaxanthin are higher when carotenoids are ingested with mono-and diacylglycerides (safflower oil) than when fat is not consumed. Similarly, White et al. [26] studied the effect of soybean oil on the absorption and bioavailability of carotenoids from spinach, lettuce, carrot, and tomato. The plasma concentrations of α-carotene, β-carotene, lutein, and lycopene increased as the concentration of soybean oil increased.

Thermal treatment affects both the cell wall and carotenoid content of plants, in turn altering their bioavailability. Aschoff et al. [27] demonstrated that the bioavailability of β-cryptoxanthin, zeinoxanthin and lutein in pasteurized orange juice is higher than in fresh orange juice. In contrast, Vimala et al. [28] evaluated carotenoid content in sweet potato undergoing different treatments (cooking, frying, oven-drying, and sun-drying). Oven-drying (50–60 °C) maintained 90% of β-carotene in sweet potato compared to the fresh product, whereas all other treatments decreased carotenoid content between 15% and 30%. Odriozola-Serrano et al. [29] examined the effect of pasteurization and electrical pulses on the carotenoid content of tomato juice. They found that tomato juice treated with electrical pulses had a higher carotenoid content. Thus, pulse treatment is the most efficient method of preserving carotenoid content and increasing their bioavailability compared to the traditional treatment. In all previously cited examples, there is a decrease in total carotenoid content; nevertheless, the bioavailability of carotenoids improves by reducing dietary fiber, releasing cellular content, softening plant material, and reducing the interactions between carotenoids and other food components. Thus, promoting both the release of carotenoids and formation of micelles helps increase their absorption.

## 5. Use of Carotenoids

Carotenoids are bioactive compounds found in fruits and vegetables. A bioactive compound is defined as a natural food ingredient of plant origin without nutritional value, which when ingested produces positive health effects, promotes health, or exerts toxic effects [30]. Carotenoids have been used as natural colorants and antioxidants due to the presence of conjugated double bonds. However, some carotenoids are a source of vitamin A, whereas others have been employed in the treatment or prevention or both of certain diseases. This section will describe some of the major uses of carotenoids.

### 5.1. Colorants

Natural colors are extracted or isolated from natural sources (plants, animals, or microorganisms) and some can be synthesized, yet all are exempt from certification as they are recognized as safe (GRAS). Carotenoids are used as natural colorants as they are derived mainly from vegetable sources and add yellow, orange, and red colors to foods. Table 1 lists carotenoids used as natural colorants, exempt from certification.

The food additive E160a, which is a mixture of carotenes or β-carotene, is obtained commercially from carrot. In contrast, the food additive E160b is obtained from the seeds of the *Bixa orellana* tree and annatto extract or the purified compounds bixin and norbixin can be used as additives. Paprika extract is obtained from bell pepper (capsicum annuum) and is composed of the carotenoids capsanthin and capsorubin. Lycopene is mainly extracted from tomato. β-Apo-8’-carotenal is a carotenoid by-product obtained from carotenes or isolated from plants. Ethyl ester of β-Apo-8′-carotenoic acid is a slightly water-soluble derivative of β-Apo-8′-carotenal. Xanthophylls such as flavoxanthin, lutein, cryptoxanthin, rubixanthin, violaxanthin and rhodoxanthin are extracted from the petals of buttercup (ranunculus sp.), tagetes sp., physalis sp., rosa rubiginosa, viola sp. and taxus baccata, respectively. Canthaxanthin is obtained from mushrooms, crustaceans, shrimp waste, and flamingo feathers, although it can also be prepared synthetically from carotene. Zeaxanthin is obtained mainly from vegetables belonging to Zea. Citranaxanthin is mainly obtained synthetically, although it can be extracted from several dried plant species. Astaxanthin is naturally extracted from krill, shrimp, crawfish, shellfish, and crustaceans and biosynthesized in microalgae (H. pluvialis). The colorant E164, saffron (crocus sativus L.), contains crocin as the main carotenoid component [14].

### 5.2. Vitamin a Activity

The nutritional value of carotenoids is primarily based on their activity as provitamin A, meaning their ability to be converted into vitamin A [13]. Provitamin A carotenoids, such as β-carotene, α-carotene, γ-carotene, and β-cryptoxanthin, are obtained from carrot, spinach, sweet potato, and orange. However, carotenoid concentration is not the only factor that determines the quality of nutritional source; bioavailability and ability to be converted into retinol must also be considered [13,33]. Furthermore, sapotexanthin, cryptocapsin, and β-Apo-8′-carotenal are also carotenoids with provitamin A potential [14,34].

Provitamin A carotenoids have a β-ionone ring, responsible for their role as retinol precursors, which is not present in other carotenoids such as lycopene, lutein, and zeaxanthin, none of which possess provitamin A activity [35].

Vitamin A exists in three forms: retinal, retinol, and retinoic acid. Vitamin A, obtained through diet, comes from plants (provitamin A carotenoids) or animals (retinol esters). After intake, retinol esters are stored in tissues, mostly liver, or converted into trans-retinaldehyde and trans-retinoic acid, by the action of alcohol and aldehyde dehydrogenases, respectively [36].

The main source of vitamin A is β-carotene as it can transform into two retinol molecules in the presence of oxygen by β-carotene 15,15′-monooxygenase [17]. This enzyme splits β-carotene into two trans-retinal molecules, which are either oxidized into retinoic acid by retinal dehydrogenase or reduced into retinol by retinal reductase [37]. In contrast, enzymatic cleavage of α-carotene and β-cryptoxanthin produces only one retinol molecule [17].

Vitamin A is essential for the development and maintenance of sight, although it plays other roles in the body, such as modulating gene expression, promoting embryonic development, reproduction, cellular growth and differentiation, strengthening the immune system, stimulating metabolic processes in the gastrointestinal tract, and reducing the risk of cancer [13,17,37] (Álvarez et al. 2015; Gul et al. 2015; Olmedilla-Alonso 2017). Table 2 shows the vitamin A requirements for men and women at various stages of life.

Retinoic acid serves as a signaling molecule in vascular development and hematopoiesis during the embryonic stage. Moreover, it is involved in the regulation and homeostasis of the immune system, differentiation of T cells, movement of T cells into tissues, development of antibody-dependent T cells, proliferation and differentiation of B cells, protection of B cells from apoptosis by binding with toll-like receptor 9, modulation of granulocyte lineage, differentiation of neutrophils, and treatment of cancer [36]. The use of trans-retinoic acid with arsenic trioxide improves the lifespan of patients with acute promyelocytic leukemia. Furthermore, trans-retinoic acid combined with interferon-α disrupts the metabolism of CD38 malignant cells, increasing their sensitivity to anti-CD38 antibodies in T cell leukemia [36].

According to Rubin et al. [39], multiple studies have established a relationship between low plasma levels of retinol and retinol-binding protein (RBP) and inflammatory processes, such as acute infections, chronic degenerative diseases, and trauma. Cser et al. [40] found that levels of β-carotene, α-carotene, β-cryptoxanthin, and retinol are lower in children with acute infections, compared to healthy children. These alterations are characterized by elevated levels of interleukin-6, which induces the expression of genes coding for acute-phase proteins, leading to decreased levels and synthesis of RBPs, thereby decreasing the intake of carotenoids and vitamin A levels and accumulation [39].

### 5.3. Antioxidants

An antioxidant delays or prevents the deterioration, damage, or destruction caused by oxidation. Antioxidants are capable of slowing, inhibiting or preventing the oxidation of molecules by quenching free radicals and stabilizing the molecules [41]. Recent studies suggest that carotenoids possess antioxidant activity, which helps protect against chronic diseases, such as cataract, coronary heart disease, certain types of cancer, obesity and asthma [7,22]. Carotenoids play a key role as electron-carriers and protectors of cells, tissues and organs from damage induced by reactive oxygen species (ROS), reactive nitrogen species (RNS), and lipid peroxides [3,42]. Free radical scavenging is carried out through electron transfer (Equations (1) and (2)), adduct formation (Equation (3)), and the transfer of hydrogen atoms (Equation (4)) [21,43].

(1) $$Car+R^{•+}\;\to Car^{•+}+R$$

(2) $$Car+e^{-}\;\to Car^{•-}$$

(3) $$Car+ROO^{•}\;\to {\left[Car-ROO\right]}^{•}+ROO^{•}\;\to ROO-Car-ROO$$

(4) $$Car\left[H\right]+R^{•}\;\to Car^{•}+RH$$

β-carotene, α-carotene, zeaxanthin, β-cryptoxanthin, lutein, and lycopene have high antioxidant activity and promote the removal of singlet oxygen (^1^O_2_), H_2_O_2_, nitric oxide radical (NO^•^), and peroxynitrite anion (ONOO^−^) owing to their isoprenoid structure (conjugated double bonds) [17,21,44]. Even though all carotenoids display antioxidant properties, lycopene, astaxanthin, and β-carotene are more effective at removing singlet oxygen compared to other carotenoids [11,43].

Numerous researchers have reported that carotenoids have antioxidant properties under both in vitro and in vivo conditions. Pons et al. [45] have shown that β-carotene, extracted from oranges, increases the resistance of the nematode caenorhabditis elegans to oxidation. You et al. [46] showed that β-carotene and synthetic carotenoids (BAS and BTS) can reduce ROS levels in *C. elegans*. Liu et al. [15] and Yazaki et al. [47] demonstrated the antioxidant capacity of astaxanthin through the increased lifespan of *C. elegans* via DAF-16 (a homolog of the mammalian transcription factor FOXO) mediated expression of catalase and superoxide dismutase (SOD), which are components of the antioxidant defense mechanism and the decreased production of mitochondrial ROS. Lashmanova et al. [48] found that fucoxanthin (0.3–10 μM) increases the longevity of and oxidative stress resistance in *C.* elegans.

The intake of antioxidants in combination is more effective than when taken alone in preventing oxidative stress. Carotenoids are a part of the organism’s antioxidant system, working in synergy with other antioxidants [43,49]. Milde et al. [50] revealed that the combination of rutin (flavonoid) and lutein or lycopene decreases LDL oxidation compared to effects achieved when antioxidants were used alone. Varakumar et al. [51] demonstrated that mango wine made from the Alphonso variety is rich in carotenoids and phenolic compounds and inhibits LDL oxidation in rats, displaying a greater effect than that achieved with other mango varieties with lower carotenoid or phenolic levels. In both studies, a clear relationship was observed between carotenoids and phenolic compounds, revealing a synergic interaction between both antioxidants. In both cases, the complex mixture of compounds in extracts or food matrix enhances the effectiveness of antioxidants by synergistic interactions between the antioxidants [45,52].

A hypercaloric diet combined with a lack of physical activity, alteration in lipid metabolism, and oxidative stress damage lead to lipid oxidation and accumulation of ROS [53,54]. As the generation of free radicals during lipid oxidation leads to cell membrane damage, LDL oxidation is a key factor in the development of both atherosclerosis and coronary heart disease [21,50]. The intake of β-carotene, astaxanthin, and lycopene prevents LDL oxidation, neutralizes peroxide radical formation, and reduces platelet aggregation [1,43]. Lutein ingestion caused a slight thickening of the carotid arteries, decreasing the risk of atherosclerosis, compared to those without a carotenoid rich diet [55].

Macular degeneration is an age-related eye disease that leads to blindness and sight loss. Evidence suggests that subjects with a low carotenoid diet and low xanthophyll levels in the retina are more prone to macular degeneration [18,55]. Lutein and zeaxanthin protect macular cells from oxidative stress and inhibit the formation of drusen (fat deposits) [1,55].

Carotenoids reduce hip fracture risk in men with osteoporosis by neutralizing oxidative stress, which plays a key role in the regulation of osteoblasts and osteoclasts activities [56].

However, some studies have suggested that a high intake of carotenoids may result in the production of pro-oxidative agents, thus harming health [21]. Ribeiro et al. [43] suggested that pro-oxidant effects often occur in cases of high oxidative stress. Similarly, Cruz-Bojórquez et al. [35] reported that animals supplemented with 30 mg β-carotene/day and exposed to cigarette smoke for six months showed a decrease in retinoic acid levels, causing precancerous cells to appear, a phenomenon not observed when lower doses (6 mg β-carotene/day) were used. The intake of β-carotene by smokers with a history of myocardial infarction increases the risk of fatal coronary diseases [48]. Chen et al. [57], Desjardins et al. [58], Jara-Palacios et al. [59] and Yazaki et al. [47] have suggested that high levels of antioxidants exhibit a pro-oxidant and toxic effect on the organism, whereas lower concentrations display a protective effect. Moreover, some breakdown products, such as epoxy-carotenoids, are harmful to the organism [43].

Pro-oxidant effects are reflected as oxidative damage to cellular structures (DNA, lipids, and proteins), overproduction of ROS, and alteration of antioxidant defense mechanisms, redox-sensitive genes and transcription factors (NF-κβ and activator protein 1) [43].

### 5.4. Biological Properties and Other Uses of Carotenoids

Despite the fact that more than 600 different types of carotenoids exist [3,60], few have significant bioactivity [61]. Those which are precursors of vitamin A have been extensively studied due to the importance of this vitamin in such essential activities as the growth and maintenance of tissues, immune response, and regeneration of photoreceptors [62]. Furthermore, the antioxidant properties of carotenoids have played a key role in cellular and molecular processes associated with the prevention of chronic diseases [13]. Carotenoids are involved in cell proliferation, signaling and communication, causing changes in transcription and protein expression. These changes have been associated with interactions between carotenoids or their derivatives (apocarotenoids and/or retinoids) and transcription factors, or through indirect modification of transcriptional activity [11,37].

Nevertheless, the most important biological activity of carotenoids is their anticarcinogenic properties and induction of apoptosis due to their antioxidant power, although beneficial properties against diabetes mellitus and cardiovascular diseases have also been reported [63,64]. The positive effect of carotenoids against several types of cancer has been previously reported, e.g., leukemia [65], colon cancer [66], prostate cancer [67], cervical cancer [68], breast cancer [69], hepatocarcinoma [70] and skin cancer [71], among others. Carotenoids have been used in cancer treatment because they regulate changes in the expression of proteins involved in cell proliferation and differentiation, apoptosis, angiogenesis, DNA repair, carcinogen elimination, and immune vigilance [11,72]. For example, β-carotene can induce the caspase cascade by interacting with the signaling complexes at the cell membrane, thereby triggering apoptosis or programmed cell death. Moreover, β-carotene can induce the release of cytochrome c from the mitochondria and alter mitochondrial membrane potential to promote apoptosis [11]. Xavier and Pérez-Gálvez [44] have reported that the intake of 50 mg of β-carotene for 10–12 years increases the activity of NK (natural killer) cells and reduces tumor occurrence. The biosynthesis of prostaglandin E2, an immune suppressor, is regulated by β-carotene, thereby strengthening the immune system. Lycopene inhibits the growth of lung cancer cells [11]. Lycopene, zeaxanthin and lutein inhibit cell proliferation in prostate and breast cancers [1], whereas capsanthin and other carotenoids isolated from *Capsicum annuum* L. have anti-tumor properties [11].

For the case of the use of carotenoids for the treatment of diabetes [13], though the action mechanism remains unclear, the antioxidant effect must have a pivotal role within the process, but other mechanisms are presumably involved [63].

Other studies suggest that a rich diet in carotenoids significantly lowers the risk of knee osteoarthritis [73], osteoporosis [74], and arthritis ([75], increased bone growth and inhibition of bone resorption [76], significant decreased risk of aged-related cataract [77,78], and enhanced risk reduction against late aged-related macular degeneration, which leads to vision loss in older adults [13,79].

The role of carotenoids in cardiovascular disease also has been studied, with presumably beneficial effects by diminishing both oxidative stress and inflammatory response [80]. Recent findings indicate a positive correlation between the concentration of serum carotenoids and better cardiovascular disease markers such as high-density lipoprotein cholesterol, triglycerides, blood insulin and fasting blood glucose, among others [81].

Interestingly, recent studies demonstrate the role of carotenoids as therapeutic agents against viral infections such as COVID-19 and HIV. In the case of their use against COVID-19, carotenoids are important as immune regulators, diminishing the inflammation caused by the activation of NF-κB and MAPK signaling pathways, which provokes an over-production of pro-inflammatory cytokines and hyperinflammation [60]. Moreover, carotenoids have been demonstrated to potentially protect against tuberculosis in patients with HIV due to their anti-inflammatory properties, although further investigation needs to be carried out [82]. A low plasma concentration of carotenoids in patients with HIV has been observed, but an alternative is the administration of β-carotene, which increases the levels of CD4+ and CD8+ lymphocytes [64,83]. As previously stated [60,84], more research must be carried out to understand the positive effect of these bioactive compounds, particularly in the treatment of emerging diseases.

## 6. Stability of Carotenoids

As carotenoids are composed of repetitive units of isoprene, which has double conjugated bonds, they are highly sensitive to light, heat, high temperatures, acids, oxygen, metals, and free radicals. This leads to structural changes (cycling, migration of double bonds and the addition of oxygen molecules) resulting in the formation of epoxy-carotenoids and apocarotenoids, compounds without biological activity [5,43,85].

Light, high temperature, oil, and dehydration cause isomerization of carotenoids and formation of 15-cis-, 9-cis- and 13-cis-β-carotene and α-carotene isomers. Cis isomers have less provitamin A potential than trans isomers, thereby decreasing the nutraceutical properties of carotenoids [5,86].

Several authors have reported the degradation of carotenoids in vegetable products exposed to thermal treatments. Addis et al. [72] showed that the carotenoid content of coccinia grandis and trigonella foenum-graecum leaves decrease upon drying (from 139.80 mg/100 g to 109.90 mg/100 g) and dehydration (from 139.80 mg/100 g to 63.20 mg/100 g and 116.60 mg/100 g to 96.20 mg/100 g, respectively). In contrast, Aschoff et al. [27] demonstrated that pasteurization of orange juice decreased its carotenoid content (230.50 μg/100 g) compared to fresh oranges (328.70 μg/100 g). Moreover, Odriozola-Serrano et al. [29] found that carotenoid levels in fresh tomato juice (14.10 mg/100 mL) decreased after pasteurization and storage at 4 °C for 56 days (7.3 mg/100 mL).

Although heat treatment lowers carotenoid content, a few carotenoids have been found to increase in concentration upon heat treatment. This is caused by enhanced product stabilization, enzymatic denaturation, and moisture loss, all of which alter the cell membrane and protein-carotenoid complexes, making carotenoids more accessible to extraction [29]. Plasma levels of β-carotene, α-carotene, β-cryptoxanthin, lutein, and zeinoxanthin were higher after the consumption of pasteurized orange juice instead of fresh oranges, increasing carotenoid bioavailability between 25% and 30% [27]. Addis et al. [72] reported that blanching (for a short time) of green leaves releases bound carotenoids and improves their extraction, thereby increasing their content compared to raw products.

The loss of carotenoids in fruit and vegetable products is mainly caused by the oxidation of highly unsaturated carotenoid structures. Oxidation can be caused by auto-oxidation (spontaneous formation of free radicals in the presence of oxygen) or photo-oxidation (caused by oxygen in the presence of light) leading to carotenoid bleaching and resulting in colorless products. Some of these breakdown products are 5,6-epoxy-β-ionone, 5,6-epoxy-β-carotene, 5,8-epoxy-β-carotene, 5,6,5′6′-diepoxy-β-carotene, 2,6,6-trimethyl-cyclohexanone, dihydroactinidiolide, β-cyclocitral, 4-oxo-β-ionone, 2-methyl-2-hepten-6-one, β-ionone, pseudo-ionone, 6-methyl-3,5-heptadien-2-one, β-carotene 5,8-endoperoxide, geranial, neral, geronic acid, acycloretinal, aurochrome and mutatochrome [29,43,86,87,88,89].

## 7. Encapsulation Techniques for Carotenoid Protection

The use of carotenoids in the food industry has been limited because of their poor stability against chemical (oxygen, metals, free radicals) and environmental (light and heat) agents, insolubility in water, high melting points and low bioavailability. The lipophilic nature of carotenoids limits their direct incorporation into aqueous systems, therefore multiple carriers have been designed such as emulsions, nanoemulsions, liposomes, hydrogel particles (a network of hydrophilic polymer trapping solvent molecules), and solid-liquid particles (crystallized lipid dispersed in oil) [17,90]. Among them, emulsions are widely used for carotenoid formulations.

Emulsions are colloidal dispersions of two immiscible liquids with one liquid dispersed (dispersed phase) into the other (continuous phase) as small droplets [91]. Emulsifying or stabilizing agents that reduce surface tension between phases are mostly used, thus facilitating emulsion formation and sustaining its integrity [92]. Stabilizing agents include small molecules such as polyoxyethylene sorbitan fatty acid esters (Tweens) or larger molecules such as proteins (casein, whey protein, soybean protein and bovine serum albumin), phospholipids (lecithin) and polysaccharides (gums, pectin, and modified starch) [93].

Emulsions are divided into water in oil emulsions (w/o), where the dispersed phase is water and the continuous phase is oil, and oil in water emulsions (o/w), where the dispersed phase is oil and the continuous phase is water [91]. O/W emulsions are prepared before carotenoid encapsulation to achieve high encapsulation efficiency and narrow particle size distribution [90,94].

To prevent the degradation of carotenoids and preserve their antioxidant activity, encapsulation techniques, which improve their stability and affect their bioavailability and solubility [95], are used. Encapsulation techniques consist of coating one or more sensitive substance (pigments, antioxidants, essential oils, and drugs), known as the core material, with another component that acts as a barrier (wall material, carrier, capsule or membrane) [6,17].

### 7.1. Microencapsulation

Microencapsulation involves covering a liquid, solid, or gas with a surrounding material (carrier) [94]. Carrier agents must protect the coated substance against environmental conditions such as moisture, heat, light, oxygen, pH, and other compounds [96]. Common carriers include polysaccharides (maltodextrin, starch, chitosan, inulin, sodium alginate, carrageenan, pectin, CMC and citrus fibers), gums (Arabic gum, Mesquite gum, Guar gum and locust bean gum), and proteins (gluten, casein, gelatin, whey protein, soy protein, albumin, milk powder and oligopeptides) [17,94,97]. The selection of a suitable carrier is based on its physical properties, such as molecular weight, melting point, solubility, viscosity, diffusivity, film-forming capacity and emulsifying properties [97]. Table S2 summarizes carriers and microencapsulation techniques used for carotenoids.

Spray drying is a widely used microencapsulation technique that involves the formation of fine particles by passing a suspension through a sprayer where compressed air heated to high temperatures flows. The flow of hot air dehydrates the particles and turns them into powder, in which the substance of interest is encapsulated [17]. Thus, stable powders (size range between 1 and 1000 μm) are obtained in a short time, at low cost and temperature, which allows the encapsulation of thermolabile compounds and stabilizes the encapsulated substance [85,98].

Freeze-drying is an encapsulation technique that involves the dehydration of a frozen material (−80 to −40 °C) under vacuum sublimation at low pressure, widely used for heat-labile bioactive compounds. This method minimizes changes associated with high temperature, although it involves a long processing time and high cost. Although freeze-drying protects carotenoids against oxidation and isomerization, it results in a large mean particle size and high porosity [90,95].

Coacervation is a microencapsulation technique that includes phase separation of a homogeneous polymer solution into a polymer-rich and a polymer-poor phase. The procedure involves emulsification, followed by phase separation, cross-linking of coacervates, and lyophilization. Phase separation is often achieved through changes in pH, which results in breaking the interaction between polymers [85,90].

Another method of microencapsulation consists of the formation of inclusion complexes with oligosaccharides and polysaccharides such as cyclodextrins, glycyrrhizic acid and arabinogalactan [99]. The inclusion complex has a hydrophobic center that interacts non-covalently with the hydrophobic structure of carotenoids and traps them inside. The use of inclusion complexes has advantages such as an improvement in chemical stability, the protection of bioactive compounds from the environment, taste modification, and controlled release [93,95]. This technique has been used for the encapsulation of lycopene, β-carotene, lutein, canthaxanthin, zeaxanthin and bixin [100].

An effective microencapsulation process depends largely on the methodology employed, which affects the moisture content, water activity, particle size, encapsulation efficiency, and the morphology of microcapsules [17]. For instance, high temperatures reduce the formation time of the membrane surrounding the encapsulated compounds, thereby preventing their retention. Moreover, high temperatures lead to a decrease in carotenoid content and isomerization [94]. Juscamaita-Fabián et al. [101] have compared the effectiveness of Arabic gum (GA) and maltodextrin (MD) in the formation of carotenoid microcapsules from the petals of *Tropaeolum majus*. Researchers achieved better carotenoid retention with GA and a drying temperature of 130 °C as higher temperatures led to higher carotenoid loss. In contrast, encapsulation efficiency can be improved by increasing the ratio of core to wall material, allowing more carotenoids to be trapped within the carrier [90]. In contrast, the degradation of carotenoids is prominent in microcapsules obtained by freeze-drying than by spray drying as the latter has a smooth and less porous structure that reduces oxygen diffusion inside the particles [102].

Microencapsulation of carotenoids enables them to be easily incorporated into food as it increases their water solubility and stability. GA/MD tropaeolum majus carotenoid particles have a solubility > 98% due to drying temperatures, resulting in the formation of powders with higher porosity that increase the surface contact between particles and water molecules [101]. De Marco et al. [103] reported a solubility of 72% for bixin extracts from annatto seeds encapsulated with GA and MD, resulting in a water-soluble powder. In contrast, Przybysz et al. [104] reported that the microencapsulation of carotenes with GA-MD improves the retention of pigments and prevents their degradation during storage (room temperature without daylight) compared to carotenes dissolved in oil (54.20% vs. 22.40%, respectively). Hojjati et al. [105] showed that encapsulation of canthaxanthin with soluble soy polysaccharide (SSPS) prevents ~60% and ~70% degradation of carotenoids at 25 °C under light and dark conditions, respectively, after 16 weeks of storage, compared to free canthaxanthin under the same conditions.

Numerous authors have reported that the antioxidant activity of encapsulated carotenoids is higher compared to that of free carotenoids, given that microcapsules enhance their stability by forming a semipermeable wall that allows the diffusion of oxygen and other reactive species into the microcapsules, where they interact with the antioxidants and are eliminated [106]. Spray drying of β-carotene emulsion with GA [107,108,109] and complex coacervation with casein (CA) and Guar gum (GG) preserves the antioxidant capacity of the β-carotene in the microcapsule. Thakur et al. [85] found that CA-GG microcapsules of β-carotene obtained by complex coacervation and freeze-drying have higher antioxidant activity than free β-carotene. Faria et al. [110] and Rodrigues et al. [106] found that β-carotene, apo-8′-carotenal, and apo-12′-carotenal GA microcapsules preserve their oxygen (^1^O_2_), peroxide radical (ROO^−^), H_2_O_2_, hydroxide radical (OH^−^), hypochlorous acid (HOCl), and peroxynitrite (ONOO^−^) scavenging properties. Boiero et al. [111] demonstrated that GA microcapsules of β-carotene (1.37 mg/mL equivalent to 0.54 μg β-carotene/mL) protect riboflavin from photodegradation in milk by 30%.

### 7.2. Nanoencapsulation

Microcapsules are relatively large (>1 μm) and their size slows down their absorption. Microcapsules are polydisperse (their size is distributed) and often thermodynamically unstable, as they tend to break down over time [112]. In contrast, nanoparticles (NPs) are thermodynamically stable or exhibit long-term kinetic stability and have a small and uniform size (<1000 nm) that guarantees the rapid release and adsorption of bioactive compounds [93]. NPs are characterized by minimal phase segregation, limited interaction between bioactive compounds and other food components, preserved bioactive properties, improved absorption and bioavailability, and reduced impact on sensory attributes [113].

Nanoencapsulation techniques have been used to protect sensitive compounds from degradation, improve their bioavailability and preserve their biological activity. Nanoencapsulation techniques include top-down and bottom-up methods for the production of NPs. Bottom-up technique includes self-organization and self-assembly of molecules through nanoprecipitation, coacervation and inclusion complexion. Top-down techniques require the use of special equipment to reduce particle size and obtain NPs. Top-down techniques include extrusion, homogenization, electrospinning/spraying and emulsification-solvent evaporation processes [114]. Nanocarriers such as nanoliposomes, nanoemulsions, and lipid-based NPs are some examples of molecules used for the protection of carotenoids [115]. Table S3 lists carotenoid NPs obtained by different nanoencapsulation techniques, along with some of their characteristics.

Nanoemulsions consist of colloidal systems made of lipid droplets, smaller than 100 nm in size, dispersed in a continuous aqueous phase [90]. They are used for the encapsulation of water-insoluble bioactive compounds. Nanoemulsions are characterized by their efficiency, low production costs, increased dispersibility, stability over long periods, and high bioavailability of active substances [17]. Droplet formation is usually a simple process, however, breaking down into smaller droplets (50–200 nm) requires additional energy, often mechanical. Nanoemulsion processes are commonly divided into high- and low-energy techniques. High-energy techniques include high-pressure homogenization, sonication, and microfluidization; low-energy techniques involve spontaneous emulsification and phase inversion temperature (PIT) [115,116].

In high-energy techniques, special equipment is used to form nanoemulsions. High-pressure homogenization consists of passing a solution through a narrow opening at high pressure (100–2000 bar) and high speed (1000 km/h). Thus, droplets are deformed and broken into smaller droplets (~50 nm). The use of ultrasound technology involves the application of high vibration ultrasonic waves that break down emulsion droplets. Microfluidization is similar to high-pressure homogenization in that an emulsion is passed through a camera at an angle of 180° at high speed, decreasing the droplet size [116].

In contrast, low-energy techniques do not require mechanical energy to break down droplets into smaller ones; rather, the chemical energy present in their components is used. Spontaneous emulsification consists of preparing an organic solution, using an oil- and a water-miscible solvent, which is added to an aqueous solution containing a hydrophilic surfactant under constant stirring to form an o/w emulsion. Afterwards, the organic phase is removed by evaporation at low pressure. In contrast, PIT does not require organic solvents and instead non-ionic surfactants, such as polyethyloxylates, are used as they are capable of modifying their affinity between oil and water according to temperature. At high temperatures, polyethyloxylates are lipophilic and form w/o emulsions, whereas at low temperatures they are hydrophilic and form o/w emulsions. During cooling, interfacial tension is minimal, which facilitates the formation of nanoemulsion. However, for this to happen, surfactant concentration must be higher than critical micellar concentration [116].

Liposomes are formed of a double lipid layer that separates an internal aqueous phase from a continuous external phase. Liposomes are formed by hydrophilic-hydrophobic interactions between amphipathic (phospholipids) and water molecules. Bioactive compounds to be encapsulated (with sizes in the nanometer to micrometer range) can be confined either inside the aqueous phase or within the lipid membrane [90]. Nanoliposomes can be produced by mechanical (ultrasonication, high-pressure homogenization) and non-mechanical methods (reversed-phase evaporation). The use of liposomes as carriers has advantages such as increased stability and efficiency; however, they also present several disadvantages such as low solubility, short half-life, and difficulties in controlling liposome size that limit their use in the food industry [115].

Lipid-based NPs are divided into solid lipid nanoparticles (SLN) and nanostructured lipid carriers (NLC). Lipid-based NPs have a lipophilic matrix structure consisting of biocompatible and biodegradable lipids, which are mainly solid at room temperature, and a surfactant and co-surfactant, which stabilize lipophilic components in the aqueous phase [93]. SLN consist of a core of solid lipid, where bioactive compounds are embedded into the lipid matrix [112], whereas NLCs are o/w emulsions in which the major portion of the lipid phase is constituted of solid lipid in combination with liquid lipid [117]. SLN presents several advantages in comparison with other carriers as they can incorporate both hydrophilic and lipophilic compounds and avoid the use of organic solvents and large-scale production. However, they have low loading capacity and stability problems caused by the rigid structure [115]. In contrast, NLC are modified SLN that retain their advantages and have better stability, higher loading capacity, and better release control [93].

Regardless of how nanoparticles are made, an aqueous colloidal suspension is obtained. Therefore, nanoencapsulation techniques are usually combined with drying techniques (freeze or spray drying) to improve their stability and to obtain NP powders [116].

### 7.3. Supercritical Fluids

Recently, supercritical fluids (SCF) have been suggested as an alternative for carotenoid encapsulation. An SCF is a dense liquid with the physicochemical properties of a gas. The use of SCF for the encapsulation of bioactive and thermolabile compounds has been adopted in the food industry as a “green technology” because they are non-toxic, can be easily removed without damaging the product, and generate many microencapsulated and nanoencapsulated products [90,95,118]. Moreover, this new approach overcomes the disadvantages of conventional encapsulation techniques such as poor control of size and morphology, loss of thermolabile compounds, low encapsulation efficiency, and yield [6,119].

Supercritical carbon dioxide (SC-CO_2_) is the most popular supercritical solvent used in the food and pharmaceutical industry due to its low critical point (T_c_ = 31.10 °C, P_c_ = 7.38 MPa), safety, low viscosity and reactivity, easy elimination, ability to inactivate microbes, relatively low cost, and better solubility of some lipophilic compounds [94]. SC-CO_2_ has enhanced the solubility of essential oils, bioactive compounds of plant origin, small molecular non-polar compounds, low molecular weight biopolymers, and low polarity lipophilic compounds [118]. Janiszewska-Turak [94] used SC-CO_2_ to encapsulate carotenoids such as astaxanthin, β-carotene, bixin, lutein, lycopene and zeaxanthin.

Supercritical encapsulation or micronization is divided into the following categories: supercritical anti-solvent or solution-enhanced dispersion by supercritical fluids (SAS/SEDS), rapid expansion of supercritical solutions (RESS), particle production from gas-saturated solutions (PGSS), and supercritical extraction from an emulsion (SFEE) [90,94].

SAS/SEDS is the most common method for micronization and consists of the use of an SCF, usually SC-CO_2_, as an anti-solvent to reduce the solubility of the active substance and encapsulating agent in their solvent and precipitate them into crystals of several morphologies (threads, sponges, leaves, needles, crystals and spheres) [94]. NPs are formed by the rapid diffusion of an organic solvent into the aqueous phase and co-precipitation of active compounds and carriers at the solvent/anti-solvent interface. Afterwards, the solvent can be removed by evaporation [90]. The main advantages of SAS/SEDS processes are related to reduced particle size due to the rapid precipitation of solutes, higher solubility rate, almost complete elimination of solvent, and the ability to encapsulate thermolabile compounds [120].

The use of SCF as anti-solvents has been explored by Xia et al. [121] and Zhao et al. [122] for the development of lutein-loaded liposomes through SAS using SC-CO_2_ as an anti-solvent. The researchers obtained liposomes with a particle size of 500 and 155 nm and encapsulation efficiency of 90 and 97%, respectively, using the following conditions: pressure, 8 MPa; temperature, 35 °C; and flow rate, 1 mL/min and pressure, 300 bar; temperature, 50 °C; and depressurization rate, 90 bar/min, respectively. In contrast, Machado Jr. et al. [123] employed SEDS to encapsulate astaxanthin in poly(hydroxybutyrate-co-hydroxyvalerate) using SC-CO_2_ as an anti-solvent. They tested different settings and found that the best encapsulation efficiency (48.25%) and smallest particle size (128 nm) were obtained at 35 °C, 100 bar, and 1 mL/min.

RESS encapsulation uses the solvation power of SCFs by adjusting pressure and temperature. The bioactive compound and the encapsulating agent are dissolved in SC-CO_2_ and precipitated by rapid depressurization and nucleation when passing through a nozzle [90,124]. RESS can be used to encapsulate a wide range of inorganic, organic and polymeric materials, operated at low-temperature, with single-step processing [118]. However, the main limitation is the low solubility of wall materials in SC-CO_2_. Therefore, RESS is rarely used for carotenoid encapsulation by SCF, and few reports have been published. At least one of these reports is from a decade ago and was produced by Quan et al. [125], who used SC-CO_2_ for astaxanthin encapsulation and obtained particles ranging from 0.3 to 0.8 μm. However, it is possible to use a liquid anti-solvent (ethanol or methanol) for the polymer as a co-solvent to overcome the drawback [6].

PGSS uses SC-CO_2_ as a solute to saturate the solution containing the bioactive. The gas-saturated solution expands at atmospheric pressure as it goes through an atomizing nozzle; gas vaporization cools the solution, promoting nucleation and precipitation of the particles [90,94].

De Paz et al. [126,127] employed PGSS and SC-CO_2_ for the microencapsulation of β-carotene. They used poly-(ε-caprolactones) as carriers to obtain particles of micrometer-scale (111–652 μm). However, when soy lecithin was used for encapsulation with the PGSS-drying technique, smaller particles (10–500 μm) were formed, which, upon rehydration, formed liposomes with a diameter of 0.9–6.1 μm.

The SFEE method employs the recrystallization of the active compound, incorporated in an o/w emulsion, using the supercritical anti-solvent process, combining the efficiency of using SFC as an anti-solvent for the formation of particles with the effect of emulsification on the formation of micro and nanoparticles (Silva and Meireles 2014) [120]. SC-CO_2_ is used to extract the organic solvent from the emulsion, leading to the supersaturation of the bioactive compound and polymer in the aqueous phase, resulting in their precipitation [118]. The presence of surface-active compounds in the emulsion promotes particle formation without agglomeration and restricting particle size due to high nucleation rates. Surfactants compatible with this technique include polysorbates (Tweens), sodium caseinate, whey proteins, β-lactoglobulin, modified starch, MD, GA and pectin [90].

SFEE has been used by Tirado et al. (2019) for the encapsulation of astaxanthin with ethyl cellulose. NPs with a size ranging from 242 to 363 nm were obtained. Santos et al. [128] used SC-CO_2_ to generate lycopene and β-carotene nanoemulsions with particle sizes between 344 and 366 nm.

Carotenoid stability in micronized particles depends on the composition and nature of carotenoids, type and concentration of lipid phase, surfactant, particle size, interfacial composition, pH, ionic strength, and environmental conditions (light, oxygen, and temperature) (Soukoulis and Bohn, 2018) [90].

Selecting the appropriate wall material is a crucial factor for supercritical encapsulation of bioactive compounds because carriers must protect the core material from environmental damage, limit the loss of volatile compounds, and allow the controlled release of the active substance [120]. In light of this, polysaccharides (gums, starches, celluloses, inulin and cyclodextrins), proteins (casein, gelatin and soy protein) and polymers (polylactic acid, polyhydroxy butyric acid and polyethylene glycol) have been used as encapsulating agents in micronization processes [95,119].

## 8. Conclusions and Future Perspectives

Carotenoids are natural hydrophobic pigments. The isoprenoid (conjugated double bonds) structure of carotenoids contributes to their color (wide range of red, orange, and yellow) and antioxidant activity. Moreover, carotenoids possess many health and nutritional properties linked to health promotion and reduced risks of many diseases. However, they are chemically unstable and prone to oxidation under various storage conditions (light, high temperature, oxygen, acid, and metal ions). To overcome this challenge, various encapsulation techniques, such as microencapsulation, nanoencapsulation and encapsulation with supercritical fluids, have been employed. Encapsulation not only enhances the stability of carotenoids and protects them from degradation but also increases their solubility in aqueous systems and bioavailability. To achieve effective encapsulation, regardless of the encapsulation technology employed, an appropriate wall material is essential for the protection and safe delivery of carotenoids. Microencapsulation is the most common method for the encapsulation of carotenoids as it employs simple encapsulation techniques and produces good quality products. However, microcapsules tend to decrease in stability over time. In contrast, nanoencapsulation technology allows for obtaining more stable products with excellent absorption and bioavailability. Multiple carriers, such as nanoemulsions, nanoliposomes, SLN, and NLC, can be used for the encapsulation of carotenoids. In contrast, alternative technologies or “green technologies,” such as supercritical encapsulation, are good alternatives for the micro and nanoencapsulation of thermolabile compounds (carotenoids) and suitable for application in the food industry without having a detrimental effect on the sensory attributes. Methods for carotenoid encapsulation presented in this review have many advantages but also some disadvantages. As carotenoids are important for the pharmaceutical and food industry, in the future it is crucial to examine encapsulation technologies focusing on their biological application as well as their interactions with other components in food systems. Moreover, it is important to evaluate their behavior in vivo rather than just addressing encapsulation efficiency, particle size or other encapsulation properties to determine the most suitable and sustainable method of encapsulation.

## Figures and Tables

**Table 1:** List of carotenoids used as food color in the European Union and United States.

Carotenoids	Food Additive	Color	Approved Use In		Food Application [14,31]
			EU [31]	USA [32]	
Carotenes or β-carotene	E160a	Yellow to Orange	Yes	Yes	Soft drinks, juice, butter, preserves of red fruits, vegetables in vinegar or brine, jam, chesses, candies, breakfast cereals, fats, sausages, pates, bakery products, precooked and smoked fish.
Carrot oil		Yellow to Orange	NR	Yes	Food generally.
Bixin and norbixin from/or Annatto extract	E160b	Orange to Brown Red	Yes	Yes	Dairy products and fermented milk, butter, fats, breakfast cereals, ice cream, desserts, custards, candies, snacks, cheeses, smoked fish, alcoholic beverages and sausages.
Capsanthin and capsorubin from/or Paprika oleoresin	E160c	Red	Yes	Yes	Breakfast cereals, cheeses, creams, sausages, surimi, preserves of red fruits, instant soups, snacks, smoked fish, pates, jams, jellies and marmalades.
Lycopene or tomato extract or tomato concentrate	E160d	Bright to Deep Red	Yes	Yes	Sauces, fermented milk products, edible ices, seafood, snacks, desserts, condiments, dietary supplements, meat substitutes, coating, fillings and decorations of bakery products, soups, chesses and flavored drinks.
β-Apo-8′-carotenal	E160e	Orange Red to Yellow	Yes	Yes	Orange and lemon soft drinks, juice, nectars, shakes, cheese, jams, jellies, marmalades and precooked food.
Ethyl ester of β-Apo-8′-carotenic acid	E160f	Orange Red to Yellow	Yes	No	Orange and lemon soft drinks, juice, nectars, shakes, margarines, butter, pies, cereals, and precooked food.
Flavoxanthin	E161a	Yellow	No	No	Candies, yoghurts and dairy products.
Lutein	E161b	Orange Red to Yellow	Yes	Yes ^a^	Jams, instant soups, creams, yoghurts, cheese, seafood, soft drinks, alcoholic beverages and poultry feed.
Cryptoxanthin	E161c	Orange to Red	No	No	Confectionary.
Rubixanthin	E161d	Orange to Red	No	No	Confectionary.
Violaxanthin	E161e	Orange	No	No	Confectionary.
Rhodoxanthin	E161f	Yellow	No	No	Confectionary and ice cream.
Canthaxanthin	E161g	Orange	No	Yes ^a^	Fish and poultry feed.
Zeaxanthin	E161h	Orange to Red	No	No	Confectionary.
Citranaxanthin	E161i	Yellow	No	No	
Astaxanthin	E161j	Red	Yes ^a^	Yes ^a^	Fish and poultry feed.
Saffron (Crocin)	E164	Gold Yellow to Orange	NR	Yes	Rice dishes, sausages, margarine, butter, cheese, ice cream, alcoholic and non-alcoholic beverages.

^a^ Only in animal feed. NR: not reported.

**Table 2:** Intake recommendations for Vitamin A [38].

Age	Recommended Dietary Allowances (µg RAE/Day)	
	Male	Female
0 to 6 months	400	400
7 to 12 months	500	500
1 to 3 years	300	300
4 to 6 years	400	400
9 to 13 years	600	600
14 to 18 years	900	700
		750 ^a^
		1200 ^b^
19 to 50 years	900	700
		770 ^a^
51+ years	900	700

RAE: retinol activity equivalents. 1 µg RAE = 1 µg retinol = 12 µg β-carotene = 24 µg α-carotene or β -cryptoxanthin. ^a^ Pregnancy. ^b^ Lactation.

## Data Availability

Not applicable.

## Supplementary Materials

The following supporting information can be downloaded at: https://www.mdpi.com/article/10.3390/plants12020313/s1, Figure S1: Chemical structure of carotenoids; Table S1: Carotenoid content in fruits and vegetables common in America; Table S2: Microencapsulation techniques and properties of encapsulated carotenoids; Table S3: Examples of carotenoids encapsulated by nanoencapsulation techniques.
