# Supplementary material for: Chemistry, Occurrence, Properties, Applications, and Encapsulation of Carotenoids—A Review
Source: Plants (Basel). 2023 Jan 9;12(2):313. doi: 10.3390/plants12020313 (PMC9865331; doi:10.3390/plants12020313)
Supplement: Supplementary file 1 [file plants-12-00313-s001.zip › Table S1.pdf]

Table S1. Carotenoid content in fruits and vegetables common in America.

Source	Scientific name	Carotenoids (μg/g)						References
		β-carotene	α-carotene	β-cryptoxanthin	Lutein	Zeaxanthin	Lycopene	
Apple								
Golden delicious	*Malus domestica*	1.40	-	-	0.71	0.02	-	[3]
Gala		0.17	-	-	0.06	0.01	-	
Apricot	*Prunus armeniaca*	170.00	-	0.73	0.09	2.70	-	[3]
Arugula	*Eruca sativa*	79.60	2.80	-	74.40	0.06	-	[3]
Banana	*Musa paradisiaca*	0.66	0.18	-	0.04	-	-	[3]
Banana	*Musa acuminata*	8.38	8.62	-	0.90	-	-	[3]
Black sapote	*Diospyros digyna*	79.90	-	-	-	-	-	[22]
Broccoli	*Brassica oleracea var. italiaca*	11.38	-	0.15	28.05	-	-	[3]
Brussels sprout	*Brassica oleracea var. gemmiera*	0.72	-	11.63	11.63	-	-	[3]
Carrot	*Daucus carota*	53.60	45.00	-	1.50	-	-	[3]
		47.5	47.1	-	-	-	-	[23]
Cashew	*Annacardium occidentale*	139.90[a]	-	-	-	-	-	[22]
Cassava	*Manihot esculenta*	7.28	0.08	0.14	0.38	-	-	[24]
Cauliflower	*Brassica oleracea var. botrytis*	0.22	-	0.15	0.28	-	-	[3]
Coriander	*Coriandrum sativum*	586.10	-	-	-	-	-	[3]
Dragon fruit	*Hylocereus undatus*	29.30[a]	-	-	-	-	-	[22]
Eggplant	*Solanum melongena*	0.18	-	-	0.71	-	-	[23]
Grape	*Vitis vinifera*	0.23	-	-	0.47	-	-	[3]
Guava	*Psidium guajava*	2.00	-	-	2.10	-	45.30	[3]

Source	Scientific name	Carotenoids (µg/g)						References
		β-carotene	α-carotene	β-cryptoxanthin	Lutein	Zeaxanthin	Lycopene	
Kale	*Brassica oleracea var. acephala*	44.00	-	0-21	65.22	-	-	[3]
Kiwifruit (golden)	*Actinidia chinensis*	11.70[a]	-	-	-	-	-	[25]
		13.90[a]	-	-	-	-	-	
Lettuce	*Lactuca sativa*	14.90	-	-	13.50	-	-	[3]
Jackfruit	*Artocarpus heterophyllus*	0.61	0.06	0.05	1.57[b]	-	-	[26]
Jocote	*Spondias purpurea*	171.60[a]	-	-	-	-	-	[22]
Mamey	*Pouteria sapota*	37.64	-	-	0.87	-	-	[4]
		361.20[a]	-	-	-	-	-	[22]
Mango	*Mangifera indica*	-	-	-	31.70	1.50	-	[3]
Melon	*Cucumis melo*							
Orange flesh		55.20	-	2.77	1.67	-	-	[27]
Green flesh		5.06	-	0.27	4.84	-	-	
Nance	*Byrsonima crassifolia*	145.40[a]	-	-	-	-	-	[22]
Okra	*Abelmoschus esculentus*	1.93	-	-	8.98	-	0.19	[23]
Orange	*Citrus x sinensis*	0.21	0.11	1.86	0.61	-	-	[28]
Papaya	*Carica papaya*	-	-	-	237.00	14.10	-	[3]
Passion fruit								
Yellow pulp	*Passiflora edulis*	13.34	0.86	2.54	0.44	0.65	-	[29]
Purple pulp		1.72	0.68	0.31	0.11	0.07	-	
Orange pulp		7.45	-	-	1.05	0.91	4.40	
Peach	*Prunus persica*	9.30	-	1.60	-	1.10	-	[3]
Pepper	*Capsicum annuum*							
Green		17.50	-	-	-	-	-	[18]

Source	Scientific name	Carotenoids (μg/g)						References
		β-carotene	α-carotene	β-cryptoxanthin	Lutein	Zeaxanthin	Lycopene	
Green		4.30	-	-	12.30	-	-	[19]
Red		16.30	-	-	3.50	-	-	[19]
Red		27.30	-	5.90	-	36.10	-	[30]
Orange		12.30	-	11.20	283.90	1513.90	-	[30]
Yellow		3.00	-	4.30	210.80	22.20	-	[30]
Pepper (Habanero)	*Capsicum chinense*							
Orange		-	-	-	6.87	0.25	-	[30]
Yellow		0.33	-	-	0.90	3.55	-	[30]
Pineapple	*Ananas comusus*	9.90	-	-	-	-	-	[3]
Pitanga	*Eugenia uniflora*	2.90	-	16.00	-	-	166.00	[20]
Plum	*Prunus domestica*	0.4	-	0.1	1.2	-	-	[19]
Pumpkin	*Cucurbita maxima*	172.20	39.90	-	-	-	-	[3]
Spinach	*Spinacia oleracea*	365.30	-	-	775.80	15.10	-	[3]
Sweet potato	*Ipomoea batatas*							[31]
Orange		365.03	16.16	-	0.91	-	-	
Yellow		117.00	8.61	-	-	5.44	-	
Purple		113.86	-	-	-	-	-	
White		90.95	3.30	-	-	20.47	-	
Tomato	*Lycopersicum sculentum*	16.10	-	-	-	-	19.40 - 46.80	[18,19]
Welsh onion	*Allium fistulosum*	29.9	-	-	48.5	-	-	[19]
Yellow corn	*Zea mays*	-	-	1.70	13.10	6.20	-	[3]
Zucchini	*Cucurbita pepo*	-	-	-	172.00	-	7.00	[3]

[a] Total carotenoid content quantified as μg of β-carotene/g. [b] Lutein + zeaxanthin content.
