# Supplementary material for: Chemistry, Occurrence, Properties, Applications, and Encapsulation of Carotenoids—A Review
Source: Plants (Basel). 2023 Jan 9;12(2):313. doi: 10.3390/plants12020313 (PMC9865331; doi:10.3390/plants12020313)
Supplement: Supplementary file 1 [file plants-12-00313-s001.zip › Table S2.pdf]

Table S2. Microencapsulation techniques and properties of encapsulated carotenoids.

Carotenoid	Emulsion composition	Method and conditions	Wall material	[EA]	Particle size (μm)	EE (%)	Storage conditions	Carotenoid retention after storage (%)	Reference
Astaxanthin	Flaxseed oil (10%), SC (10%), lactose (10%) and water (70%)	Spray drying at 180 °C	SC-lactose (1:1)	NR	6 to 100	86.06 ± 1.44	26 days at 5, 25 and 40 °C	73.83 (5°C)<br>60.17 (25°C)<br>47.09 (40°C)	[87]
Astaxanthin	Sunflower oil and EA solution (1:4)	Spray drying at 160, 170 and 180 °C	SCF-WPI (1:1)<br>SCF-SC (1:1)	30%<br>30%	NR<br>NR	~ 95.00	NR	NR	[88]
α-carotene and β-carotene	Rapeseed oil (5%), EA (30%) and water (65%)	Spray drying at 190 °C	GA-MD (1:1)	30%	NR	59.40	12 weeks at 20°C without access to daylight	54.20	[89]
β-carotene	Oil (10%), EA (25%) and water (65%)	Spray drying at 190 °C	GA-MD-MS (1:1:1)	25%	NR	88.60 ± 0.40	64 days at 20°C under daylight	75.70	[90]
β-carotene	β-carotene (0.05%) and GA solution (99.95%)	Spray drying at 170 °C<br>Drum drying at 140 °C<br>Freeze drying	MD	40%	30 ± 10<br>105 ± 15<br>80 ± 25	38.00 ± 2.00[a]<br>66.00 ± 2.00[a]<br>50.00 ± 2.00[a]	15 weeks at 25, 35 and 45 °C and RH of 11 and 32%	~ 27.00<br>> 47.00<br>~ 38.00	[91]
β-carotene	Dichloromethane (carotenoids) and EA solutions	Spray drying at 170 °C	GA	20%	10.7 ± 0.2	99.00	NR	NR	[92]
			MD	20%	7.1 ± 0.1	63.90			
Apo-8′-carotenal			GA	20%	9.4 ± 0.1	96.60			
			MD	20%	6.4 ± 0.1	75.70			
Apo-12′-carotenal			GA	20%	8.9 ± 0.1	94.90			
			MD	20%	7.4 ± 0.2	92.80			
β-carotene	Soya oil (1%), EA solution (99%)	Complex coacervation and freeze drying	CA-GG (1:0.05)	NR	170.76 ± 4.87	57.05 ± 6.64	Intense exposure to UV rays for 5 days	NR	[74]
			CA-GG (1:0.1)	NR	180.43 ± 3.09	62.60 ± 3.68			
			CA-GG (1:0.25)	NR	165.83 ± 5.93	63.16 ± 1.04			
			CA-GG (1:0.5)	NR	176.47 ± 4.65	65.95 ± 5.33			
β-carotene and lutein	Sunflower oil, WPI solution and EA solution	Spray drying of SL emulsions at 185 °C	Trehalose-MD (1:1)	57.14%	92.27 ± 0.64	NR	92 days at 25, 45 and 65 °C	NR	[93]
		Spray drying of LBL emulsions at 185 °C	Trehalose-MD (1:1) and GA	57.14% and 2.91%	107.30 ± 0.40	NR			
	Same composition as SL and LBL emulsions	Freeze drying	Same composition as SL and LBL emulsions	-	NR	NR			
Banana passion fruit (*Passiflora mollissima*)	-	Spray drying at 120 °C	MD	3%	NR	NR	NR	NR	[94]

Bixin	Annatto extract and EA (1:4)	Spray drying at 180 °C	GA-MD (1:1)	NR	NR	75.69	18 days at 25°C under ligh and dark conditions	NR	[95]
Canthaxanthin	Corn oil and EA solution (1:4)	Spray drying at 170 °C	SSPS	10%	7.94 ± 0.14	90.30 ± 0.70	16 weeks at 4, 25 and 45 °C under light and dark conditions	75(D) and 58(L) at 25°C<br>95(D) and 88(L) at 4°C	[96]
			GA	10%	9.08 ± 0.07	86.90 ± 0.30		72(D) and 53(L) at 25°C<br>93(D) and 86(L) at 4°C	
			MD	10%	10.42 ± 0.14	73.50 ± 0.60		57(D) and 26(L) at 25°C<br>84(D) and 74(L) at 4°C	
Lutein	Ethanol (lutein 10%), soybean phospholid and EA solution	Spray drying at 170 °C	Gelatin-starch (1:8)	1:10	NR	62.00 – 94.40	30 days at room temperature under daylight	> 32.20	[97]
		Spray drying at 180 °C		1:20	NR				
		Spray drying at 190 °C		1:30	NR				
Lutein and zeaxanthin	-	Inclusion complex	Glycyrrhizic acid and AG	NR	NR	NR	NR	NR	[98]
Lutein from *Tagetes erecta*	Sunflower oil, SC, inulin and water	Spray-drying at 140, 160 and 180 °C	Inulin	1:1, 1:2 and 1:3	NR	78.00 – 86.40	NR	NR	[86]
Lycopene	Acetone (lycopene) and EA solution	Spray drying at 170, 180, 190 and 210 °C	Gelatin-sucrose at multiple ratios	NR	NR	12.10 – 82.20	4 weeks at 0°C	NR	[99]
Lycopene	Dichloromethane (lycopene) and EA solution	Spray-drying at 170 °C	GA-sucrose (8:2)	20%	NR	94.00 – 96.00	NR	NR	[100]
	Dichloromethane (lycopene) and β-CD solution with Tween 20 (0.2%)	Inclusion complex and freeze drying	β-CD	1:4	NR	~ 50.00			
Paprika oleoresin	Oleoresin and MS (1:4)	Spray drying at 180 °C	MS	0.3 g/L	NR	NR	35 days at 35°C and a_w=0.108, 0.318, 0.515 and 0.743	NR	[101]
Petals of *Tropaeolum majus*	Olive oil (carotenoids), SC, water and EA solution	Spray-drying at 130 °C	MD	20%	NR	54.82 ± 0.03[a]	NR	NR	[102]
			GA	20%	NR	83.76 ± 0.06[a]			
		Spray-drying at 150 °C	MD	20%	NR	49.71 ± 0.08[a]			
			GA	20%	NR	74.10 ± 0.01[a]			
		Spray-drying at 170 °C	MD	20%	NR	45.16 ± 0.02[a]			
			GA	20%	NR	68.96 ± 0.02[a]			
Probiotic juice rich in lycopene and β-carotene	-	Spray drying between 120 and 160 °C	MD	10 to 15%	NR	NR	NR	NR	[103]

AG: arabinogalactan. CA: Casein. EA: encapsulating agent. EE: encapsulation efficiency. GA: Arabic gum. GG: Guar gum. LBL: layer-by-layer. MD: maltodextrin. MS: modified starch. NR: not reported. SC: sodium caseinate. SCF: soluble corn fiber. SL: single layer. SSPS: soluble soybean polysaccharide. WPI: Whey protein isolate. β-CD: β-cyclodextrin. [a] Carotenoid retention (%).
