# Supplementary material for: Chemistry, Occurrence, Properties, Applications, and Encapsulation of Carotenoids—A Review
Source: Plants (Basel). 2023 Jan 9;12(2):313. doi: 10.3390/plants12020313 (PMC9865331; doi:10.3390/plants12020313)
Supplement: Supplementary file 1 [file plants-12-00313-s001.zip › Table S3.pdf]

Table S3. Examples of carotenoids encapsulated by nanoencapsulation techniques.

Carotenoid	Deliver system	Surfactant	Encapsulation method	Particle size (nm)	EE (%)	Storage conditions	Carotenoid retention after storage (%)	Reference
Astaxanthin	o/w emulsion (corn oil)	SC	Microfluidization (12,000 psi 5 cycles)	230.62 ± 1.34	79.80 ± 3.10	15 days at different temperatures, pH and [NaCl] under light and dark conditions	NR	[113]
Astaxanthin	Liposome	SPC and cholesterol	High intensity ultrasound (100 W for 4 min in ice)	80.62 ± 4.52	97.68 ± 0.35	15 days at 4 and 25 °C under dark conditions	82.29 (4°C)<br>61.32 (25°C)	[114]
Astaxanthin	Liposome	SPC and cholesterol	High intensity ultrasound (100 W for 4 min in ice)	80.31 ± 1.80	97.49 ± 0.27	ND	ND	[114]
Astaxanthin	NLC: lipid phase (astaxanthin, GB, lecithin and oleic acid)	Tween 80 and lecithin	High intensity ultrasound	94	~ 90.00	Different pH, [NaCl] and temperature conditions	ND	[115]
β-carotene	o/w emulsion (corn oil)	Tween 20	Microfluidization (4,000 psi 3 times)	210 (small)<br>380 (medium)	ND	ND	ND	[116]
β-carotene	o/w emulsion (palm oil)<br>o/w emulsion (coconut oil)<br>o/w emulsion (fish oil)<br>o/w emulsion (corn oil)	WPI	Microfluidization (15,000 psi 7 cycles)	168.43 ± 7.68<br>172.70 ± 1.91<br>184.97 ± 0.86<br>176.80 ± 0.44	NR<br>NR<br>NR<br>NR	6 weeks at 25 and 55 °C under dark conditions	69.36 (25°C), 48.56 (55°C)<br>63.81 (25°C), 43.41 (55°C)<br>49.58 (25°C), 29.35 (55°C)<br>54.91 (25°C), 33.60 (55°C)	[117]
β-carotene	o/w emulsion (corn oil)	Tween 20	High-pressure homogenization (172 MPa 8 times)	173	ND	ND	ND	[118]
β-carotene	o/w emulsion (corn oil)	Tween 80	High-pressure homogenization (100 MPa 3 cycles)	226.00 ± 7.00	ND	ND	ND	[116]
β-carotene	Mix of alcoholic (zein and β-carotene) and aqueous (surfactants) solutions	Zein, Tween 80 and pluronic F-68	Phase separation and freeze drying	81.30 ± 4.20 (zein 1 mg/mL)<br>92.30 ± 3.60 (zein 2 mg/mL)<br>131.70 ± 6.10 (zein 3 mg/mL)	55.30 ± 1.90 (zein 1 mg/mL)<br>68.20 ± 2.30 (zein 2 mg/mL)<br>68.80 ± 3.10 (zein 3 mg/mL)	ND	ND	[119]
β-carotene	SLN: lipid phase A (β-carotene, GMS and gelucire 50/13) and lipid phase B (phospholipid S-100)	GMS, Tween 80 and phospholipid S-100	Homogenization (30 min at 10,000 rpm) combined with freeze drying	203.00 ± 7.23	68.30 ± 3.40	3 months at 5, 25 and 40 °C	> 60	[120]
β-carotene	o/w emulsion	BSA		744.00 ± 22.00	90.10 ± 1.20		~ 40	[121]

	o/w emulsion	GA	High-pressure homogenization (1st stage 70 Mpa, 2nd stage 7 MP 3 cycles)	199.00 ± 6.00	95.10 ± 1.50	5 weeks at 25°C under dark conditions	~ 45	
	o/w emulsion	BSA-GA		221.00 ± 5.00	94.40 ± 0.70		~ 80	
Carotenoids	o/w emulsion in CS solution with TPP (crosslinking agent)	Tween 80	Homogenization-ionic gelation (5000 rpm for 30 min) and freeze drying	317	89.09	ND	ND	[122]
Carrot puree (α- and β-carotene)	o/w emulsion (corn/olive oil)	Tween 80	High-pressure homogenization (100 MPa 3 cycles)	279.00 ± 1.00 (olive oil)<br>249.00 ± 28.00 (corn oil)	ND	ND	ND	[116]
Crocin	o/w emulsion	Alginate and CS	High-intensity ultrasound (20 kHz for 3 min on ice) and ionic gelation	236	38.16	ND	ND	[123]
Lutein	CS, DS and lutein solution mixed with PEG400 and EDC (co-crosslinking agent)	PEG400	Polyelectrolyte complexation and homogenization (15 min at 8,000 rpm)	428.00 ± 1.00 (no P/E)<br>454.00 ± 7.00 (with P/E)	60.71 ± 0.55 (no P/E)<br>76.17 ± 1.30 (with P/E)	4 weeks at 4°C (R) and room temperature (RT)	76.90 ± 22.40 (R-no P/E)<br>12.50 ± 2.00 (RT-no P/E)<br>87.10 ± 15.90 (R-with P/E)<br>11.30 ± 1.20 (RT-with P/E)	[124]
Lutein	o/w emulsion	WPI	High-intensity ultrasound (amplitude 40% for 5 min on ice)	209.00 ± 9.10	ND	4 weeks at 5, 25 and 37 °C	~ 95	[125]
Lycopene	Mix of lipid phase (lycopene, GPS or GB) and aqueous phase	Tween 80 and Poloxamer 407	Homogenization (12,000 rpm for 30 min at 70°C, 19,000 rpm for 10 min)	125.00 ± 3.89 (GPS)<br>129.00 ± 1.83 (GB)	98.40 ± 0.50 (GPS)<br>93.10 ± 0.60 (GB)	3 months at 4°C	ND	[126]
Lycopene	Liposome	Phospholipids	NR	ND	71.90	3 h at 37°C under different pH (6.5, 7.4, 8.0 and 9.0)	58.20 ± 0.40 (6.5)<br>53.90 ± 0.00 (7.4)<br>44.40 ± 0.20 (8.0)<br>20.90 ± 0.17 (9.0)	[127]
Paprika oleoresin	o/w emulsion (canola oil and oleoresin)	Tween 20	Microfluidization (30 MPa 11 cycles) combined with nano spray drying (110°C)	ND	98.50 ± 2.10	30 days at 25, 35 and 45 °C under a_w=0.108, 0.318, 0.529 and 0.743	50 (25°C and a_w=0.529)	[128]
Paprika oleoresin	o/w emulsion	Tween 40	High intensity ultrasound (250 W, 24kHz, amplitude 65% for 10 min on ice)	39.00 ± 0.03	ND	ND	ND	[129]

BSA: bovine serum albumin. CS: chitosan. DS: dextran sulfate. GB: glyceryl behenate. GMS: glyceryl monostearate. GPS: glyceryl palmitostearate. EE: encapsulation efficiency. PBS: phosphate buffer solution. ND: not determined. NLC: nanostructured lipid carrier. NR: not reported. SCP: soybean phosphatidyl coline. SLN: solid lipid nanoparticles. TPP: tripolyphosphate. WPI: whey protein isolate.
