# Supplementary figures and images for: Chemistry, Occurrence, Properties, Applications, and Encapsulation of Carotenoids—A Review
Source: Plants (Basel). 2023 Jan 9;12(2):313. doi: 10.3390/plants12020313 (PMC9865331; doi:10.3390/plants12020313)

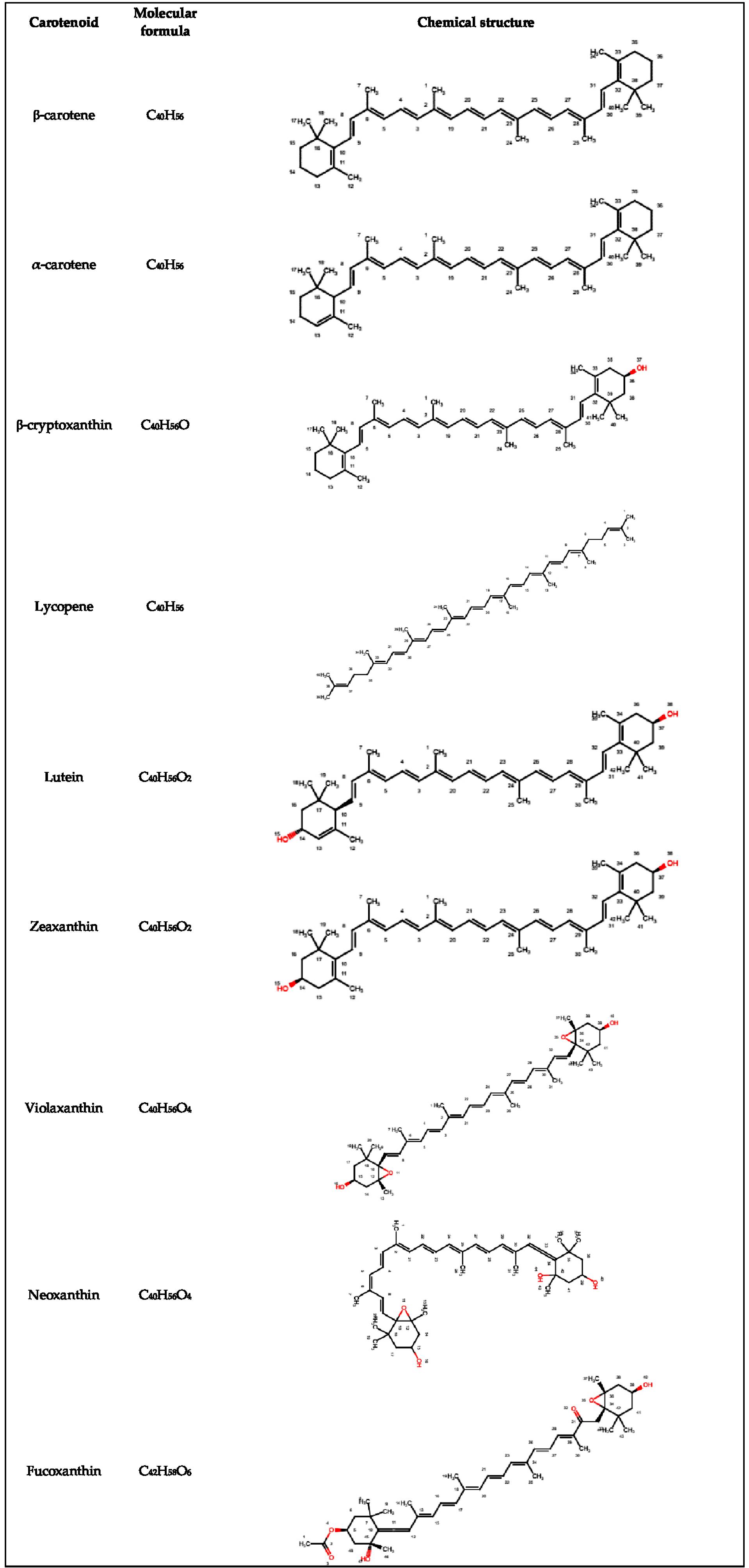

Supplement: Supplementary file 1 [file plants-12-00313-s001.zip › Fig.S1 R1 (part 1).jpg]

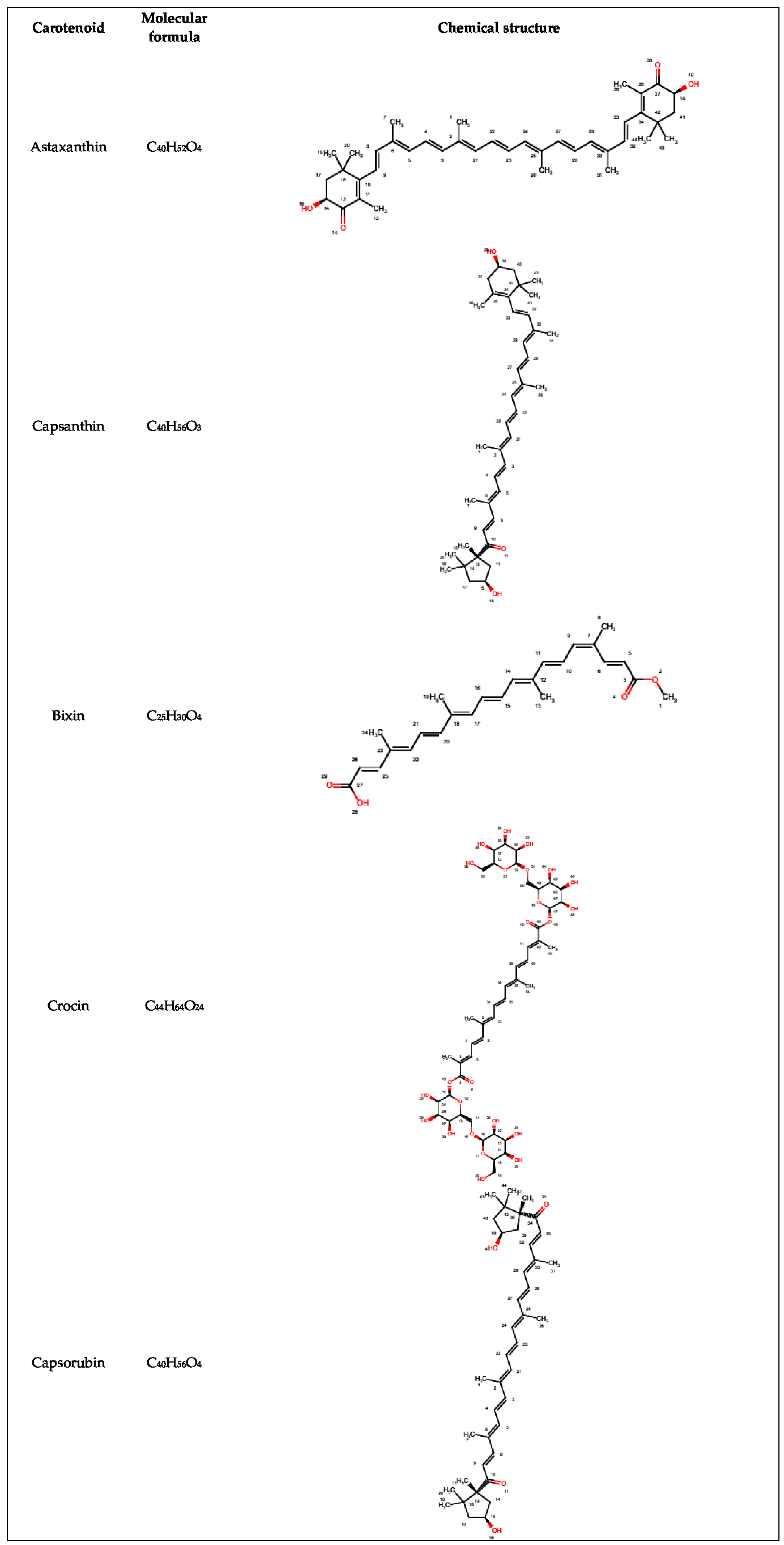

Supplement: Supplementary file 1 [file plants-12-00313-s001.zip › Fig.S1 R1 (part 2).png]

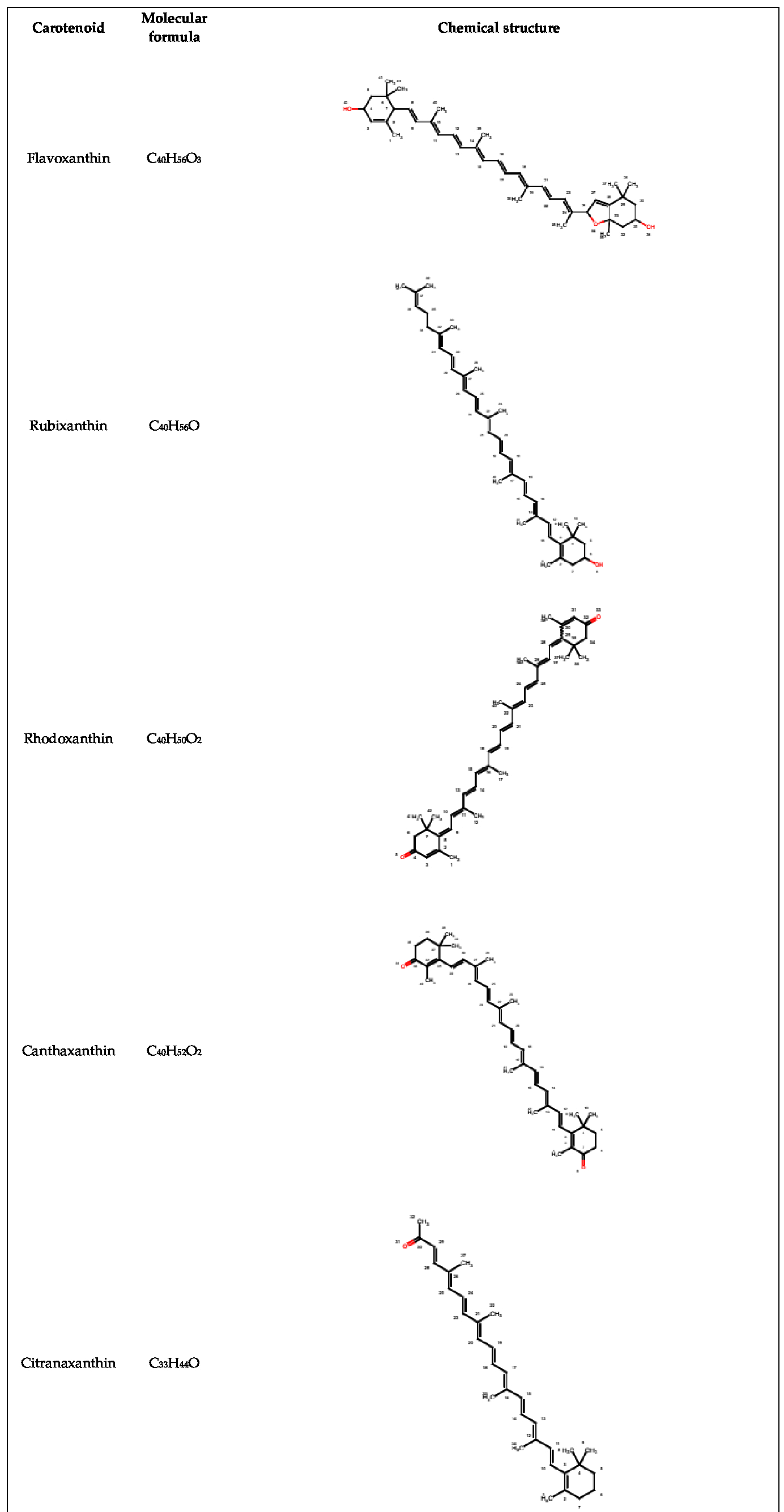

Supplement: Supplementary file 1 [file plants-12-00313-s001.zip › Fig.S1 R1 (part 3).png]
